# Drivers and recent trends of hospitalisation costs related to acute pulmonary embolism

**DOI:** 10.1007/s00392-024-02437-y

**Published:** 2024-04-02

**Authors:** Katharina Mohr, Lukas Hobohm, Klaus Kaier, Ioannis T. Farmakis, Luca Valerio, Stefano Barco, Christina Abele, Thomas Münzel, Thomas Neusius, Stavros Konstantinides, Harald Binder, Karsten Keller

**Affiliations:** 1 Center for Thrombosis and Hemostasis (CTH), University Medical Center of the Johannes Gutenberg-University Mainz, Mainz, Germany; 2 Institute of Medical Biometry and Statistics, Faculty of Medicine and Medical Center, University of Freiburg, Freiburg, Germany; 3 Department of Cardiology, University Medical Center of the Johannes Gutenberg-University Mainz, Mainz, Germany; 4 Department of Angiology, University Hospital Zurich, Zurich, Switzerland; 5 German Center for Cardiovascular Research (DZHK), Partner Site Rhine Main, Mainz, Germany; 6 Wiesbaden Business School, RheinMain University of Applied Sciences, Wiesbaden, Germany; 7 Department of Cardiology, Democritus University of Thrace, Alexandroupolis, Greece; 8 Medical Clinic VII, Department of Sports Medicine, University Hospital Heidelberg, Heidelberg, Germany

**Keywords:** Pulmonary embolism, Economic burden, Hospitalisation costs, Cost of illness, Catheter-directed treatment

## Abstract

**Background and aims:**

The socio-economic burden imposed by acute pulmonary embolism (PE) on European healthcare systems is largely unknown. We sought to determine temporal trends and identify cost drivers of hospitalisation for PE in Germany.

**Methods and results:**

We analysed the totality of reimbursed hospitalisation costs in Germany (G-DRG system) in the years 2016–2020. Overall, 484 884 PE hospitalisations were coded in this period. Direct hospital costs amounted to a median of 3572 (IQR, 2804 to 5869) euros, resulting in average total reimbursements of 710 million euros annually. Age, PE severity, comorbidities and in-hospital (particularly bleeding) complications were identified by multivariable logistic regression as significant cost drivers. Use of catheter-directed therapy (CDT) constantly increased (annual change in the absolute proportion of hospitalisations with CDT + 0.40% [95% CI + 0.32% to + 0.47%]; *P* < 0.001), and it more than doubled in the group of patients with severe PE (28% of the entire population) over time. Although CDT use was overall associated with increased hospitalisation costs, this association was no longer present (adjusted OR 1.02 [0.80–1.31]) in patients with severe PE and shock; this was related, at least in part, to a reduction in the median length of hospital stay (for 14.0 to 8.0 days).

**Conclusions:**

We identified current and emerging cost drivers of hospitalisation for PE, focusing on severe disease and intermediate/high risk of an adverse early outcome. The present study may inform reimbursement decisions by policymakers and help to guide future health economic analysis of advanced treatment options for patients with PE.

**Graphical Abstract:**

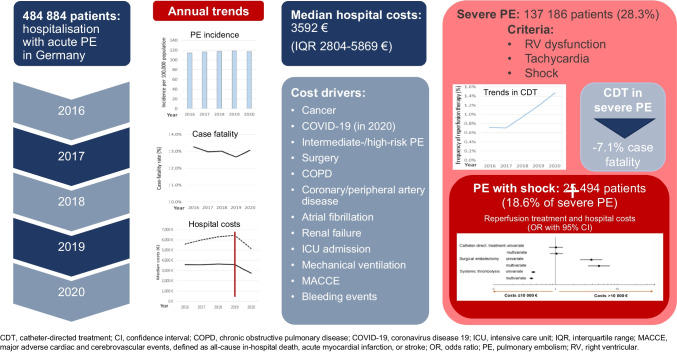

**Supplementary Information:**

The online version contains supplementary material available at 10.1007/s00392-024-02437-y.

## Introduction

Venous thromboembolism and particularly acute pulmonary embolism (PE) are a leading cardiovascular cause of death after myocardial infarction and stroke [1] and have a substantial impact on the morbidity and mortality of the population in Europe and globally [2–4]. In recent years, significant progress was made in the advanced reperfusion treatment of intermediate-risk and high-risk PE, which presents with acute right ventricular (RV) pressure overload and is a potentially life-threatening condition [5–7]. Data available so far suggest that recently introduced catheter-directed treatment (CDT) options for PE may improve survival and reduce complications and the length of hospital stay [8, 9]. However, healthcare systems in many countries remain reluctant to reimburse the costs of these emerging treatment options. Besides the need for more robust evidence on the clinical benefits of novel approaches [5, 10], there is a lack of systematically collected large-scale data on actual hospitalisation costs and the main cost drivers. In fact, a recent comprehensive analysis that provided an update on the economic burden of cardiovascular diseases across the European Union (EU) focused on coronary heart disease and cerebrovascular disease but did not mention any data related to acute PE [11].

Aiming to contribute to the closure of this gap and to begin to inform decision-making, we analysed, in the present study, the entire German nationwide inpatient population hospitalised for acute PE from 2016 to 2020. We aimed to obtain actual reimbursed hospitalisation costs, comparing them to those of the other two most frequent acute cardiovascular syndromes, myocardial infarction and (ischaemic) stroke. We further aimed to identify patient characteristics, procedures and complications independently affecting the cost of hospital stay and calculate annual trends of hospital reimbursements. In this regard, we placed a focus on patients in the upper categories of clinical PE severity and early risk of death.

## Methods

### Data source and ethical aspects

Data, including hospital reimbursements in euros, were obtained from the Research Data Center (RDC) of the Federal Statistical Office of Germany (Wiesbaden, Germany; source: RDC of the Federal Statistical Office and the Statistical Offices of the federal states, DRG Statistics 2016–2020; own calculations) as previously described [9, 12]. The authors generated SPSS codes (IBM Corp. Released 2011; IBM SPSS Statistics for Windows, version 20.0. IBM Corp: Armonk, NY, USA). These codes were submitted to the RDC, which performed the calculations on behalf of the authors and provided aggregated and summarised statistics [12]. There was neither commercial support nor external influence during the planning and performing of the analyses or the preparation of the manuscript. Since the investigators and authors of the present study had no direct access to individual patient data but used aggregated data provided by the RDC, there was, in accordance with German law, no requirement for obtaining patient informed consent or for approval by ethics committees [12].

### Coding of diagnoses and procedures

Since 2004, coding of patient data on diagnoses, coexisting conditions and surgical or other interventional procedures according to the German Diagnosis-Related Groups (G-DRG) system is required to obtain reimbursement for provided health services; for this purpose, coded data are transferred to the Institute for the Hospital Remuneration System. For this purpose, patients’ diagnoses are coded according to the International Statistical Classification of Diseases and Related Health Problems (of the 10th revision with German modification, ICD-10-GM), and diagnostic/interventional/surgical procedures are coded according to special OPS codes (*Operationen- und Prozedurenschlüssel*). We identified all patient admissions to German hospitals in the period 2016 through 2020, with PE (ICD-10 code I26) as the main or secondary diagnosis. For comparison of costs with myocardial infarction and ischaemic stroke, we used the ICD codes I21/I22 and I63, respectively. Severe PE was defined as tachycardia (I47 and R00.0), RV dysfunction (I26.0) or shock (R57). Haemodynamic instability was defined as shock (R57) or cardiopulmonary resuscitation (OPS code 8–77). The following reperfusion treatment procedures were included in the analysis: systemic thrombolysis (8-020.8), surgical embolectomy (5-380.42) and catheter-directed thrombolysis or mechanical thrombectomy (8-838.d0, 8-838.50, 8-838.60, 8-838.70, and 8-83b.j).

### Study outcomes

The primary outcome was the reimbursed cost of PE-related hospitalisation in euros. Secondary outcomes were length of hospital stay and in-hospital death (case fatality) from any cause. Further outcomes included major in-hospital adverse cardiac and cerebrovascular events (MACCE), defined as all-cause in-hospital death, acute myocardial infarction [ICD-10 code I21] or ischaemic stroke [I63]. In addition, we evaluated major bleeding including at least one of the following: gastrointestinal bleeding (K92.0, K92.1, K92.2), intracranial bleeding (I61), spinal cord haemorrhage (G95.10), haemarthrosis (M25.0), haemopericardium (I31.2) and/or necessity of transfusion of erythrocyte concentrates (OPS code 8-800).

### Statistical analysis

Continuous variables are given as medians with interquartile range (IQR), or as means ± standard deviation (SD); categorical values are given as percentages. PE patients were stratified by hospitalisation costs, using the overall median reimbursement as the cutoff value; they were also stratified by survival status (in-hospital death versus survival to discharge). Differences between groups were calculated with the Wilcoxon-Mann-Whitney *U* test for continuous variables and with chi-square or Fisher’s exact test for categorical variables as appropriate. Temporal (annual) trends of hospitalisations, costs, percentage of hospitalisations in which CDT was used, length of hospital stay and in-hospital mortality were estimated using linear regression analysis. The results are presented as beta (β) estimates with the corresponding 95% confidence intervals (CI).

Univariate and multivariable logistic regression models were constructed for investigating associations of (i) patient characteristics, comorbidities, treatments and in-hospital adverse events, with (ii) hospitalisation costs above versus below 10,000 euros. This cutoff was chosen to discriminate between patients with a less complicated hospital course and those necessitating advanced reperfusion procedures for acute PE, based on current reimbursement rates in the G-DRG system. For the multivariable regression analysis, three progressive adjustment models were used:

- Model I: adjustment for age and sex
- Model II: in addition to age and sex, adjustment for comorbidities (obesity, diabetes mellitus, cancer, coronary artery disease, heart failure, chronic obstructive pulmonary disease, essential arterial hypertension, acute and chronic kidney failure, surgery, chronic anaemia, atrial fibrillation/flutter) and for severity of PE as defined above
- Model III: in addition to age, sex, comorbidities and severity of PE, adjustment for the length of in-hospital stay

The results are presented as odds ratios (OR) with the corresponding 95% CI; *P* values < 0.05 (two-sided) were considered to be statistically significant. Associations, in specific patient subgroups (such as those with severe PE or shock), of different reperfusion treatment procedures with in-hospital mortality, major bleeding, hospitalisation costs and length of stay, are presented as forest plots showing the results of univariate and multivariable (model II) regression analysis. All statistical analyses were performed with the SPSS software (IBM Corp. Released 2011. IBM SPSS Statistics for Windows, version 20.0. IBM Corp: Armonk, NY, USA).

## Results

### Patient characteristics and costs of hospitalisation

A total of 484,884 hospitalisations of patients with acute PE (median age 71.0 [IQR 59.0 to 80.0] years; female sex 51.0%) were coded in Germany in the years 2016 through 2020, corresponding to an annual incidence of 117 PE-related hospitalisations per 100,000 population. Actual hospital costs amounted to a median of 3572 (2804 to 5869) euros, resulting in total hospital reimbursements of 3.53 billion euros throughout the 5-year period. Put into perspective, per-patient costs for acute PE were lower than those for acute myocardial infarction (4714 [3166 to 6586] euros) and ischaemic stroke (5257 [3725 to 7258] euros). The overall annual hospitalisation costs for PE averaged 710 million euros in Germany, compared to 2.02 billion euros for myocardial infarction and 2.36 billion euros for ischaemic stroke.

The patients’ demographic characteristics, medical history, clinical findings and in-hospital clinical course are presented, stratified by median hospitalisation costs, in Table 1; in Supplementary Table S1, patients are stratified by survival status. Overall, severe PE, defined by RV dysfunction, tachycardia or shock, was present in 28.3% of the cases, and 9.1% of the patients were haemodynamically unstable; treatment in an intensive care unit (ICU) was documented in 19.0% of the cases. The median length of in-hospital stay was 8.0 (IQR 4.0–14.0) days, and 62 996 (13.0%) patients died in hospital.

**Table 1:** Characteristics, treatment and in-hospital course of patients with pulmonary embolism during the years 2016–2020 in Germany, stratified by hospitalisation costs (median reimbursement amount used as cutoff)

Parameters	Costs ≤ 3600 € (*n* = 258,149; 53.2%)	Costs > 3600 € (*n* = 226,735; 46.8%)	*P* value
Age, *median (IQR)*	70.0 (58.0–80.0)	72.0 (61.0–80.0)	< 0.001
Age ≥ 70 years	133,980 (51.9%)	127,911 (56.4%)	< 0.001
Female sex	130,237 (50.5%)	117,080 (51.6%)	< 0.001
Cardiovascular risk factors			
Obesity	21,163 (8.2%)	23,906 (10.5%)	< 0.001
Essential arterial hypertension	114,235 (44.3%)	111,218 (49.1%)	< 0.001
Dyslipidaemia	34,209 (13.3%)	35,526 (15.7%)	< 0.001
VTE risk factors			
Cancer	38,700 (15.0%)	63,659 (28.1%)	< 0.001
Surgery	104,713 (40.6%)	168,465 (74.3%)	< 0.001
Pregnancy	369 (0.14%)	218 (0.09%)	< 0.001
Comorbidities			
Coronary artery disease	28,366 (11.0%)	35,988 (15.9%)	< 0.001
Heart failure	48,190 (21.6%)	64,700 (28.5%)	< 0.001
Peripheral artery disease	5532 (2.1%)	9128 (4.0%)	< 0.001
Atrial fibrillation/flutter	26,956 (10.4%)	41,978 (18.5%)	< 0.001
Chronic obstructive pulmonary disease	19,888 (7.7%)	25,562 (11.3%)	< 0.001
Acute or chronic renal failure	46,390 (18.0%)	70,025 (30.9%)	< 0.001
COVID-19	1681 (0.6%)	1681 (0.7%)	< 0.001
Diabetes mellitus	40,674 (15.8%)	49,246 (21.7%)	< 0.001
Anaemia	13,691 (5.3%)	30,293 (13.4%)	< 0.001
Clinical findings			
Severe pulmonary embolism	**66,009 (25.6%)**	**71,177 (31.4%)**	** < 0.001**
Tachycardia	6340 (2.5%)	10,797 (4.8%)	< 0.001
RV dysfunction	60,068 (23.3%)	55,474 (24.5%)	< 0.001
Haemodynamic instability	16,162 (6.3%)	28,058 (12.4%)	< 0.001
Shock	7074 (2.7%)	18,420 (8.1%)	< 0.001
Cardiopulmonary resuscitation	12,725 (4.9%)	15,735 (6.9%)	< 0.001
Treatment			
Admission to intensive care unit	22,414 (8.7%)	69,900 (30.8%)	< 0.001
Mechanical ventilation	3348 (1.3%)	15,618 (6.9%)	< 0.001
Systemic thrombolysis	9758 (3.8%)	10,384 (4.6%)	< 0.001
Catheter-directed treatment	130 (0.1%)	1607 (0.7%)	< 0.001
Surgical embolectomy	5 (0.001%)	602 (0.3%)	< 0.001
Adverse events during hospitalisation			
MACCE	**31,661 (12.3%)**	**47,824 (21.1%)**	** < 0.001**
In-hospital mortality	27,851 (10.9%)	35,145 (15.4%)	< 0.001
Stroke	3690 (1.4%)	12,900 (5.7%)	< 0.001
Pneumonia	56,379 (21.8%)	73,984 (32.6%)	< 0.001
Acute renal failure	10,940 (4.2%)	35,278 (15.6%)	< 0.001
Major bleeding	**8574 (3.3%)**	**53,311 (23.5%)**	** < 0.001**
Intracerebral bleeding	665 (0.3%)	2794 (1.2%)	< 0.001
Gastrointestinal bleeding	2067 (0.8%)	6644 (2.9%)	< 0.001
Haemarthrosis	26 (0.01%)	11 (0.03%)	< 0.001
Transfusion of blood constituents	6336 (2.5%)	48,795 (21.5%)	< 0.001
Duration of hospitalisation			
Days in hospital, *median (IQR)*	6.0 (3.0–8.0)	14.0 (8.0–22.0)	< 0.001
Hospitalisation > 7 days	84,149 (32.6%)	170,211 (75.1%)	< 0.001
Hospitalisation > 10 days	36,835 (14.3%)	138,654 (61.2%)	< 0.001

*COVID-19* coronavirus disease 19, *IQR* interquartile range, *MACCE* major adverse cardiac and cerebrovascular events, defined as all-cause in-hospital death, acute myocardial infarction or stroke, *PE* pulmonary embolism, *RV* right ventricle, *VTE* venous thromboembolism

### Variables associated with elevated hospitalisation costs for pulmonary embolism

**Fig. 1:**
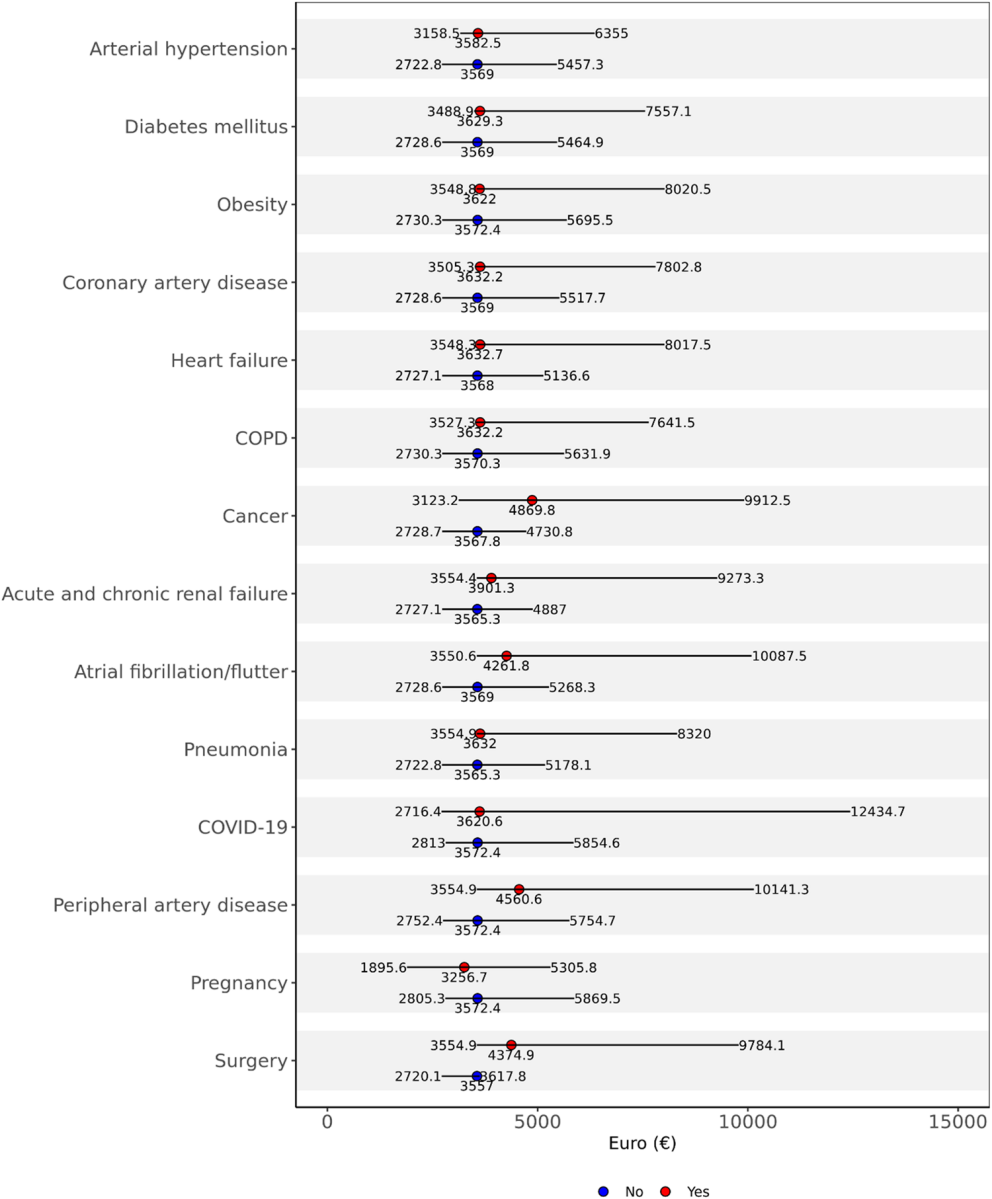
Patient characteristics as potential cost drivers during hospitalisation. For every influencing factor, median hospitalisation costs with interquartile range for presence (upper forest plot with red medians) or absence (lower forest plots with blue medians) of these factors was calculated. COPD, chronic obstructive pulmonary disease; COVID-19, coronavirus disease 19

As shown in Table 1, patients with treatment costs above the cutoff of 3600 euros (the rounded median amount of hospital reimbursement) were older and presented with an aggravated comorbidity profile. Median hospitalisation costs of patients with versus those without major risk factors and comorbidities are shown in Fig. 1. Hospitalisation costs increased in parallel to the severity of comorbidity as reflected by the Charlson comorbidity index (Fig. S1).

Patients with elevated treatment costs more frequently presented with severe PE and haemodynamic instability. Consequently, they more frequently needed admission to the ICU and advanced (reperfusion) treatment of PE, suffered a variety of in-hospital adverse cardiovascular events and other complications including major bleeding and had a more than twice as long (14.0 versus 6.0 days) duration of hospitalisation (Table 1). Logistic regression analysis, applying three models with escalating adjustment as described in the “Methods” section, identified several baseline parameters and complications conditionally associated with high in-hospital costs > 10,000 euros (Table 2). Among the variables tested, the highest ORs were obtained for cancer, concomitant coronavirus disease (COVID)-19 for the year 2020, surgery during the hospital stay, severe PE, need for admission to an intensive care unit (ICU) and mechanical ventilation, MACCE and bleeding complications. The cost of illness related to acute PE was higher at hospitals in urban compared to suburban and rural areas (see Fig. S2), probably due to the early transfer of severely ill patients with acute PE to tertiary urban hospitals and the availability of advanced options for thrombus removal from the lungs and mechanical circulatory support in the large referral centres. Mean and median hospitalisation costs per federal state (Bundeland) are displayed in Fig. S3.

**Table 2:** Parameters associated with hospitalisation costs in excess of 10,000 €

	Univariate regression	Multivariable regression (adjustment level I)	Multivariable regression (adjustment level II)	Multivariable regression (adjustment level III)
	OR (95% CI)	OR (95% CI)	OR (95% CI)	OR (95% CI)
Age ≥ 70 years	0.83 (0.82–0.84)	**0.85 (0.84–0.86)**	**0.63 (0.62–0.65)**	**0.61 (0.59–0.62)**
Female sex	0.83 (0.82–0.85)	**0.85 (0.84–0.86)**	**0.90 (0.88–0.92)**	**0.85 (0.83–0.87)**
CCI severity class	1.93 (1.91–1.95)	**−**	**−**	**−**
Cancer	2.44 (2.40–2.49)	**2.44 (2.39–2.48)**	**2.15 (2.11–2.19)**	**1.97 (1.93–2.02)**
Surgery	15.76 (15.27–16.27)	**15.91 (15.41–16.43)**	**13.38 (12.96–13.82)**	**5.85 (5.65–6.06)**
Pregnancy	0.68 (0.52–0.89)	**0.64 (0.49–0.83)**	**0.66 (0.49–0.87)**	**0.54 (0.38–0.77)**
Coronary artery disease	1.48 (1.45–1.51)	**1.52 (1.49–1.56)**	**1.07 (1.04–1.09)**	**1.10 (1.07–1.14)**
Heart failure	1.74 (1.71–1.77)	**1.85 (1.82–1.89)**	**1.25 (1.23–1.28)**	1.02 (0.99–1.05)
Peripheral artery disease	2.06 (1.98–2.14)	**2.08 (2.01–2.17)**	**1.41 (1.35–1.47)**	**1.24 (1.18–1.31)**
Atrial fibrillation/flutter	2.28 (2.24–2.33)	**2.517 (2.47–2.57)**	**1.85 (1.81–1.90)**	**1.47 (1.43–1.51)**
COPD	1.51 (1.47–1.54)	**1.53 (1.49–1.56)**	**1.38 (1.34–1.42)**	**1.20 (1.16–1.24)**
Acute or chronic renal failure	2.35 (2.31–2.39)	**2.64 (2.60–2.69)**	**1.86 (1.82–1.90)**	**1.52 (1.48–1.56)**
COVID-19	2.19 (2.03–2.36)	**2.15 (1.99–2.32)**	**3.68 (3.34–4.06)**	**2.39 (2.13–2.67)**
Diabetes mellitus	1.54 (1.51–1.57)	**1.59 (1.56–1.62)**	**1.15 (1.13–1.18)**	**1.03 (1.00–1.06)**
Anaemia	2.69 (2.63–2.75)	**2.74 (2.68–2.81)**	**1.64 (1.60–1.68)**	**0.93 (0.90–0.96)**
Severe pulmonary embolism	1.95 (1.92–1.98)	**1.97 (1.94–2.00)**	**1.99 (1.95–2.03)**	**2.13 (2.08–2.18)**
Admission to ICU	13.39 (13.15–13.64)	**13.35 (13.12–13.60)**	**11.30 (11.06–11.54)**	**8.10 (7.91–8.30)**
Mechanical ventilation	9.88 (9.59–10.19)	**9.75 (9.45–10.04)**	**6.94 (6.69–7.19)**	**3.73 (3.58–3.90)**
MACCE	3.10 (3.05–3.16)	**3.28 (3.22–3.34)**	**2.57 (2.52–2.63)**	**3.64 (3.547–3.736)**
Major bleeding	14.90 (14.61–15.19)	**15.17 (14.87–15.46)**	**9.53 (9.31–9.76)**	**5.57 (5.42–5.72)**
Intracerebral bleeding	7.44 (6.95–7.95)	**7.35 (6.87–7.87)**	**6.48 (5.99–7.00)**	**3.37 (3.06–3.71)**
Gastrointestinal bleeding	3.51 (3.35–3.67)	**3.56 (3.41–3.72)**	**1.88 (1.79–1.98)**	**1.11 (1.04–1.18)**
Transfusion of blood constituents	16.74 (16.40–17.08)	**17.05 (16.71–17.40)**	**10.99 (10.72–11.27)**	**6.56 (6.37–6.75)**
Length of hospital stay (days)	1.15 (1.15–1.15)	**1.15 (1.15–1.15)**	**1.12 (1.12–1.13)**	**1.12 (1.12–1.12)**
Hospitalisation > 10 days	19.47 (19.01–19.93)	**21.92 (21.39–22.46)**	**12.04 (11.74–12.35)**	-

Adjustment level I: adjusted for age and sex

Adjustment level II: in addition to age and sex, adjusted for comorbidities (obesity, diabetes mellitus, cancer, coronary artery disease, heart failure, chronic obstructive pulmonary disease, essential arterial hypertension, acute and chronic kidney failure, surgery, chronic anaemia, atrial fibrillation/flutter) and for severity of PE as defined above

Adjustment level III: in addition to age, sex, comorbidities and severity of PE, adjusted for the length of in-hospital stay

*CCI* Charlson comorbidity index, *CI* confidence interval, *COPD* chronic obstructive pulmonary disease, *COVID-19* coronavirus disease 19, *ICU* intensive care unit, *MACCE* major adverse cardiac and cerebrovascular events, defined as all-cause in-hospital death, acute myocardial infarction or stroke, *OR* odds ratio

### Procedures, outcomes and hospitalisation costs in patients with severe pulmonary embolism

Severe PE, with or without haemodynamic instability, was documented in 137,186 (28.3%) of the hospitalised patients. As expected, case fatality was higher (27.2% vs. 7.4%; *P* < 0.001) in this patient group compared to patients without severe PE (also see Table S1). Tables S2, S3, and S4 display outcomes and hospitalisation costs of patients with severe PE, stratified by systemic thrombolysis, surgical embolectomy and CDT, respectively. Odds ratios with the corresponding 95% CI, calculated by univariate and multivariable (adjustment level II as defined in the “Methods” section) logistic regression analysis, are displayed in Fig. S4.

Systemic thrombolysis was administered to 16,050 (11.7%) of patients with severe PE. Intravenous thrombolytic treatment was associated with a shorter duration of hospitalisation and no increase in hospitalisation costs; at the same time, however, it was associated with higher case fatality and an elevated risk for major bleeding complications. Surgical embolectomy was performed in only a small minority (504; 0.4%) of the cases. CDT, used in 1381 cases (1.0%) in Germany during the study period, was associated with higher hospital costs and more frequent major bleeding complications; on the other hand, patients who underwent CDT less frequently needed hospitalisation for a period longer than 7–10 days. Use of CDT was also associated with a lower risk of early death (OR 0.82 [95% CI 0.71–0.93]), independently from age, sex and comorbidities (Fig. S3, panel A).

**Fig. 2:**
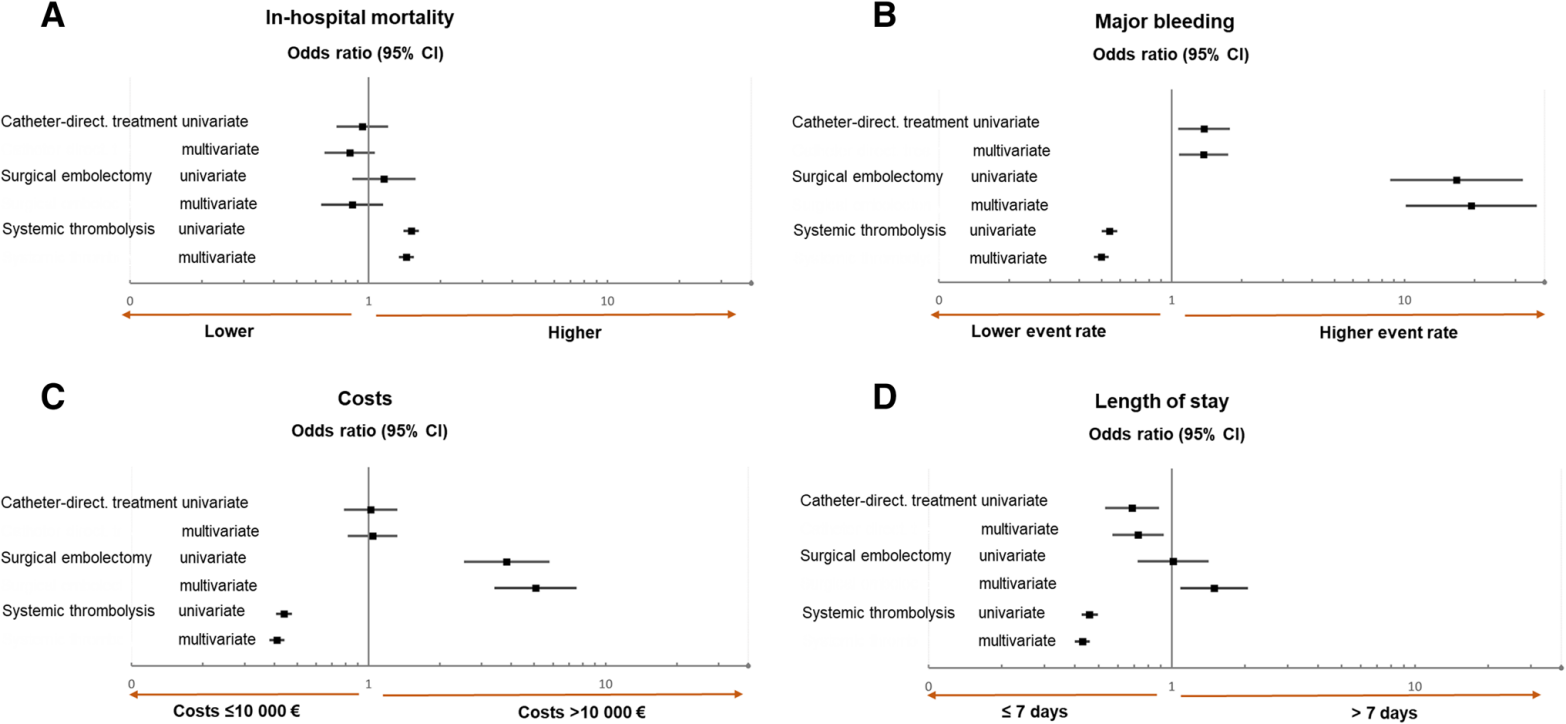
Association, in patients with shock, of different reperfusion treatment procedures with in-hospital mortality (**A**), major bleeding (**B**), reimbursed hospitalisation costs (**C**) and length of hospital stay (**D**). Results of univariate and multivariable logistic regression analysis are presented as odds ratios with corresponding 95% confidence intervals (CI), representing the use versus non-use of the respective treatment. The multivariable model adjusted for the following variables: age, sex, obesity, diabetes mellitus, cancer, coronary artery disease, heart failure, chronic obstructive pulmonary disease, essential arterial hypertension, acute/chronic kidney failure, surgery, chronic anaemia and atrial fibrillation/flutter

Patients with obstructive shock represent the highest-risk subgroup among those with severe PE [6]. In the present study, shock was documented in 25,494 (5.26%) of all hospitalised patients, corresponding to 18.6% of those with severe PE. Case fatality was considerably higher (60.6% vs. 10.3%; *P* < 0.001) in this patient group compared to patients without shock. Tables S5, S6, and S7 compare outcomes and hospitalisation costs of patients with acute PE and shock, stratified by systemic thrombolysis, surgical embolectomy and CDT, respectively. Treatment-related OR calculated by univariate and multivariable (adjustment level II) logistic regression analysis is displayed in Fig. 2. Systemic thrombolysis was administered to 5954 (23.4%) patients, and its association with the length of hospital stay and in-hospital mortality (Table S5) was in the same direction as in the entire group of patients with severe PE. At the same time, patients who underwent thrombolysis had lower major bleeding rates than those who did not. The length of stay was significantly shorter (median, 8.0 versus 14.0 days) in patients who underwent CDT, in line with the trend in the entire patient group with severe PE. Of note, overall hospitalisation costs were not increased among shock patients who received CDT compared to those who did not (Fig. 2C).

### Temporal trends of costs, treatments and outcomes

**Fig. 3:**
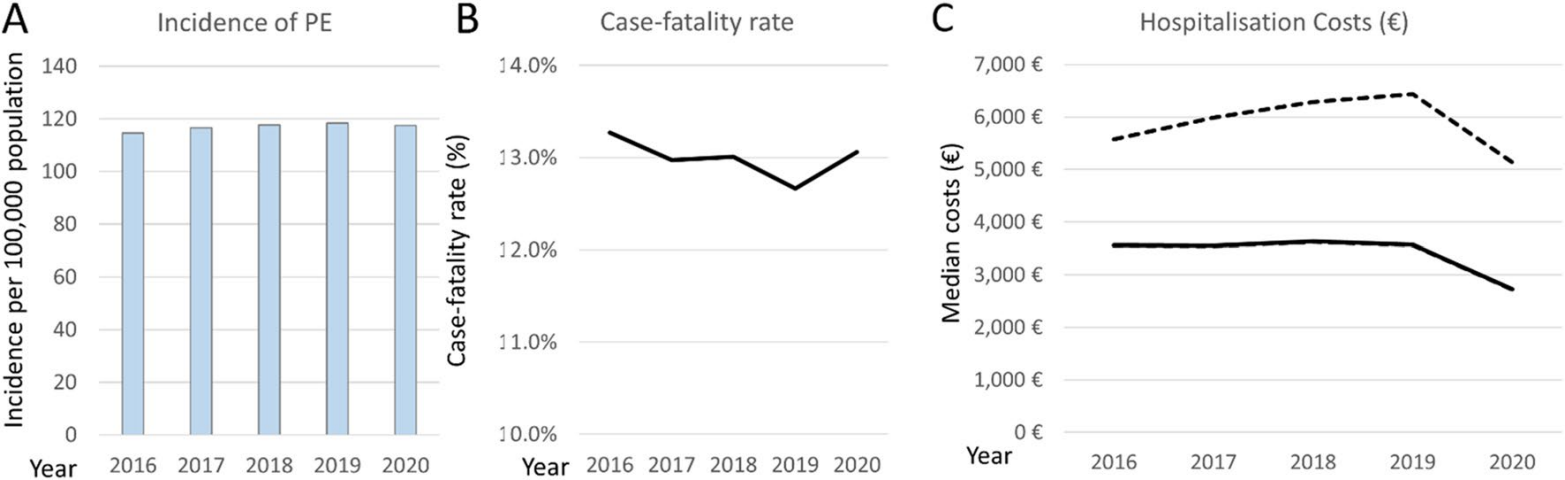
Annual trends of hospitalisations (**A**), in-hospital case fatality (**B**) and reimbursed costs **(C)**, in euros, of patients with pulmonary embolism in Germany during the study period. In **C**, the continuous black line denotes median costs; the dashed lines, the corresponding upper and lower quartile values. Note, the line of the lower quartile is almost superimposed on the solid line of the median costs

Annual hospitalisations related to acute PE in Germany increased from 94,568 (corresponding to 114.5 hospitalisations per 100,000 population) in the year 2016 to 97,718 (117.5 per 100,000) in the year 2020 (Fig. 3A); linear regression analysis revealed a significant trend for the annual increase in PE hospitalisations (*β* 0.075 [95% CI 0.070 to 0.079]; *P* < 0.001). During the same period, in-hospital case fatality decreased from 13.3% in 2016 to its lowest level of 12.7% in the year 2019, but increased again to 13.1% in the COVID-19 pandemic year 2020 (Fig. 3B). The patients’ sex distribution and comorbidity profile remained largely unchanged (not shown). The proportion of hospitalisations longer 10 days decreased over time (*β* for the annual change in absolute proportion, − 6.7% [− 7.5 to − 5.9%]). Median hospital costs in euros changed only minimally, from 3563 (3557–5572) in 2016 to 3572 (3564–6438) in 2019, and dropped to 2725 (2721–5134) in the year 2020 (Fig. 3C).

**Fig. 4:**
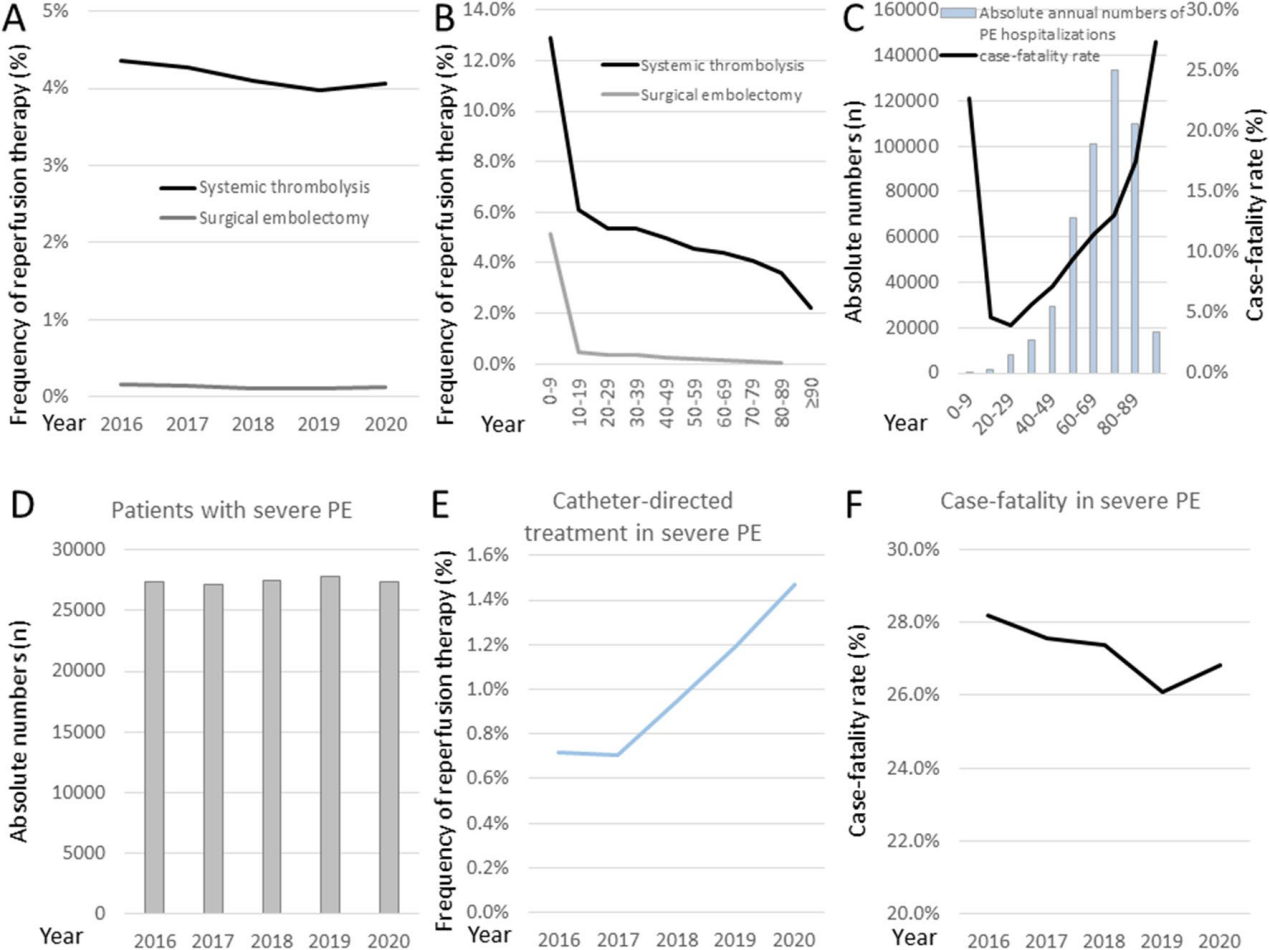
The changing landscape of pulmonary embolism management in Germany. Annual trends revealed progressive decline of the proportion of reperfusion treatment with systemic thrombolysis (*β* for annual absolute change, − 0.04% [95%CI − 0.06 to − 0.02%]) and a constantly very low rate of surgical embolectomy in the entire population of hospitalised PE patients (**A**). Frequency of use of systemic thrombolysis and surgical embolectomy was relatively high (only) among very young patients, but it fell with growing age (**B**). It is however in older patients, after the sixth decade of life, that absolute numbers of hospitalisations and case fatality rates of PE increased dramatically (**C**). Annual trends further showed consistently large numbers of hospitalisations with severe pulmonary embolism (**D**). The proportion of use of catheter-directed treatment hospitalisations of patients with severe pulmonary embolism increased constantly since 2017 (**E**). Finally, linear regression analysis showed a decrease in case fatality of severe pulmonary embolism, with a (possibly temporary) rebound in the pandemic year 2020 (**F**)

Analysis of annual trends revealed a progressive decline of reperfusion treatment with systemic thrombolysis and a constantly very low rate of surgical embolectomy in the entire population of hospitalised PE patients (Fig. 4A). Throughout the study period, the frequency of systemic thrombolysis and surgical embolectomy was relatively high only among very young patients and fell rapidly with growing age (Fig. 4B). These findings support the need for alternative options of reperfusion treatment in adult, mainly older patients. Indeed, after the sixth decade of life case fatality rates rose dramatically (Fig. 4C), while the annual numbers of admissions with severe PE remained consistently high (Fig. 4D). As shown in Fig. 4E, there was a constant increase in CDT use between 2017 and 2020 (annual change in the absolute proportion of hospitalisations during which CDT was performed: + 0.40% [95% CI + 0.32 to + 0.47%]). In parallel to this trend, our analyses revealed an overall decrease in case fatality of severe PE, with a (presumably temporary) rebound in the pandemic year 2020 (Fig. 4F).

## Discussion

The present study investigated the economic burden of acute pulmonary embolism, including all patients hospitalised with PE in in Germany, the country with the largest population in Europe (currently 84 million), over 5 years. Our results can be summarised as follows: (1) actual median hospitalisation costs in Germany were higher than those estimated from European registry data, albeit lower than the costs related to myocardial infarction and (ischaemic) stroke; (2) age, PE severity and comorbidity, as well as in-hospital (particularly bleeding) complications, were identified by multivariate logistic regression as significant cost drivers in the study population; (3) use of CDT doubled over time in the elevated-risk group of patients with “severe” PE (28% of the entire population) and (4) catheter-directed procedures, although costly by themselves as reflected by current reimbursements, were not associated with an overall increase in hospitalisation costs among patients with severe PE and shock. A reduced length of hospital stay paralleled this latter finding.

Very little is known about the actual hospitalisation costs imposed by acute PE on national health systems. A recent cost-of-illness analysis, based on data from 1349 patients diagnosed with PE and included in a prospective European registry, provided a low-end cost estimate of 2328 euros and a high-end estimate of 3533 euros for the hospitalisation related to the index acute event [13]. Earlier models analysing cost sources from several European studies had yielded higher median estimates, between 3891 and 4197 euros [14]. Such approximations may have limitations given the relatively small, heterogeneous patient populations analysed; moreover, several adjustments and assumptions must be made to account for the differences in healthcare systems across Europe [13, 14]. By comparison, in the present study, we analysed actual documented and reimbursed hospitalisation costs in the entire population of a single country. Our results in a population of almost half a million patients in Germany revealed median hospitalisation costs of 3572 euros during the study period, lying at the upper end of the most recent European estimate mentioned above [13]. On the other hand, the cost of illness of acute PE in Germany remained, at least over the years studied, substantially lower than the costs reported for PE-related hospitalisations in the US which reached a median of 10,032 (IQR 4467 to 20,330) US dollars in the years 2016–2018 [15].

Time trend analysis revealed that annual hospitalisation costs for acute PE remained relatively stable during the study period, during which no new major diagnostic or therapeutic procedures for this disease (demanding substantial changes in reimbursement) entered broad clinical practice. Interestingly, a remarkable drop in reimbursed costs occurred in the year 2020. This may be related, at least in part, to the observed increase of in-hospital case fatality of PE associated with COVID-19, particularly in the first year of the pandemic [16–18], in some cases reducing the duration of hospitalisation. In parallel to a change in the natural history of PE itself, the lockdown measures imposed in Germany and other countries significantly impacted hospitalisations for a broad spectrum of potentially life-threatening diseases, including severe oncological and cardiovascular cases and also emergencies such as acute myocardial infarction [19–25]. This was due to the combination of reduced hospital capacities in view of the large number of beds being reserved for COVID-19 cases and the patients’ fear of contracting the infection by seeking medical help and being admitted to a hospital. It is thus likely that an undetermined number of patients with severe PE never reached the hospital during that period. Moreover, some cases of severe PE may have gone unnoticed in hospitalised patients, their critical condition being attributed to COVID-19 if the virus test was positive. Finally, the reduction of elective surgical procedures during the pandemic, and consequently of PE cases associated with them, may have contributed to this temporary reduction in reimbursements.

A contemporary topic of debate is to what extent new catheter-directed interventions for dissolving or fragmenting/aspirating pulmonary emboli may impact healthcare costs in Europe and other parts of the world in the coming years. CDT systems are undergoing continuous technical evolution and are increasingly popular among physicians in the US [26], particularly at institutions that have established multidisciplinary pulmonary embolism teams (PERT) [27, 28]. In Europe, the introduction of CDT was initially more hesitant [9], but its use has now begun to increase as well as confirmed by the results of our study. For example, modelling recent CDT trends in the US, we could calculate the expected increase of CDT use in Germany for the future period 2025–2030. Our models predict a CDT penetration ranging from 3.1 to 8.7% by 2030, resulting in an increase of annual costs for PE-related hospitalisations between 15.3 and 49.8 million euros [29].

Observational data suggest that CDT, when used in patients with intermediate-risk or high-risk PE, may reduce the risk of bleeding complications, and it may also be associated with lower in-hospital mortality [8, 9, 30]. Nevertheless, current guidelines demand convincing high-quality data from randomised controlled trials before endorsing CDT as first-line therapy in patients without haemodynamic collapse [5, 6, 10]. In addition, direct costs of catheter systems and procedures for advanced PE therapy need to be determined separately for each country’s hospital reimbursement system and, importantly, be weighed against their benefits in terms of reduced early complications, length of hospital and ICU stay, return to work and productivity and prevention of late sequelae of PE [31]. In agreement with previous reports [8, 9, 30], we observed a significant association between CDT and a reduction of in-hospital mortality and the length of hospital stay. Although CDT use was, as expected, associated with higher hospital costs in the entire group of patients with severe PE, this was no longer the case when the subgroup of high-risk patients with shock was analysed. These findings generate the hypothesis that the cost–benefit ratio of CDT might become increasingly favourable with increasing severity of PE.

Some limitations exist and caution is warranted when attempting to translate associations found in an analysis of nationwide data into possible causal relationships between treatment modalities and hospital outcomes, complications and/or costs. Firstly, neither the physicians’ rationale regarding patient selection for individual therapeutic measures nor the exact timing of complications during the hospital stay, particularly in relation to systemic thrombolysis or advanced interventional procedures, can be retrieved from this type of aggregated observational data. A typical example of this limitation is our finding that patients with PE and shock who underwent thrombolysis appeared to have major bleeding less frequently than those who did not, seemingly contradicting the established bleeding risk of this treatment form [32]. This may be due to the fact that patients with an excessive bleeding risk (which is often the case in this risk category) do not receive this type of treatment in clinical practice [6] but bleed nevertheless, even on heparin anticoagulation alone; besides, patients with shock have a high in-hospital case fatality, and major bleeding occurring immediately before death may have been underreported.

Secondly, due to the nature of an ICD- and OPS-code-based analysis, classification of the severity of PE in the present study may not exactly correspond to the definition proposed by European guidelines [6]. Besides, underreporting of adverse events and/or undercoding of procedures cannot be excluded. However, it is very unlikely that costly complications and treatments were ‘forgotten’, considering the reimbursement efforts of all involved hospitals. Thirdly, we cannot exclude an interdependence of patients’ comorbidities which were included in the progressive adjustment models of our multivariable regression analysis. Finally, no follow-up evaluation after hospital discharge was available. Ongoing randomised trials will help to determine not only the efficacy and safety of CDT techniques compared to medical treatment but also if their implementation on a larger scale and across different healthcare reimbursement systems is cost-effective [33].

In conclusion, the present study provided actual cost-of-illness data for the entire population hospitalised for acute PE in a large European country over a 5-year period. Our results help to identify current and emerging cost drivers. They may inform reimbursement decisions by policymakers and help to guide future health economic analysis of advanced treatment options for patients with intermediate-and high-risk PE.

## Supplementary Information

Below is the link to the electronic supplementary material.

Supplementary file1 (DOCX 839 KB)

## Data Availability

The statistical analysis for this study was carried out on the authors’ behalf by the Research Data Center (RDC) of the Federal Bureau of Statistics, Wiesbaden, Germany, including the entire nationwide inpatient population of Germany for the years studied (source: RDC of the Federal Statistical Office and the Statistical Offices of the federal states, DRG Statistics 2020, own calculations). All codes used in this study are publicly available online. The data are aggregated analysis results provided by the RDC; this means that the authors had access to summarised results provided by the RDC, but no access to individual patient-level data, which are not publicly available.
