# Supplementary material for: Drivers and recent trends of hospitalisation costs related to acute pulmonary embolism
Source: Clin Res Cardiol. 2024 Apr 2;114(10):1246–57. doi: 10.1007/s00392-024-02437-y (PMC12460522; doi:10.1007/s00392-024-02437-y)
Supplement: Supplementary file 1 — Supplementary file1 (DOCX 839 KB) [file 392_2024_2437_MOESM1_ESM.docx]

**Online Supplementary Material**

**Recent Trends and Drivers of Hospitalisation Costs Related to Acute Pulmonary Embolism**

**Running head: Cost of Illness Acute PE**

**Katharina Mohr, MA^1,2*^; Lukas Hobohm, MD^1,3*^; Klaus Kaier, PhD^2^, Ioannis T. Farmakis, MD^1,3^; Luca Valerio, MD, PhD^1^; Stefano Barco, MD, PhD^1,4^; Christina Abele, MSc**^1^**; Thomas Münzel, MD^3,5^; Thomas Neusius, PhD**^6^**; Stavros Konstantinides, MD^1,7^; Harald Binder, PhD^2#^; Karsten Keller, MD^1,3,8#^**

**Affiliations:**

^1^ Center for Thrombosis and Hemostasis (CTH), University Medical Center of the Johannes Gutenberg-University Mainz, Mainz, Germany

^2^ Institute of Medical Biometry and Statistics, Faculty of Medicine and Medical Center, University of Freiburg, Freiburg, Germany

^3^ Department of Cardiology, University Medical Center of the Johannes Gutenberg-University Mainz, Mainz, Germany

^4^ Department of Angiology, University Hospital Zurich, Zurich, Switzerland

^5^ German Center for Cardiovascular Research (DZHK), Partner Site Rhine Main, Mainz, Germany

^6^ Wiesbaden Business School, RheinMain University of Applied Sciences, Wiesbaden, Germany

^7^ Department of Cardiology, Democritus University of Thrace, Alexandroupolis, Greece

^8^ Medical Clinic VII, Department of Sports Medicine, University Hospital Heidelberg, Heidelberg, Germany

* The first two authors contributed equally to the study.

**^#^** The last two authors contributed equally to the study.

**Table S1 Characteristics, treatment and in-hospital course of patients with pulmonary embolism during the years 2016-2020 in Germany, stratified by** **survival to discharge from hospital**

| **Parameters** | **Patients who survived to discharge**  **(*n*=421 888; 87.0%)** | **Patients who died in hospital**  **(*n*=62 996; 13.0%)** | **p value** |
| --- | --- | --- | --- |
| Age, *median (IQR)* | 71.0 (59.0-79.0) | 76.0 (65.0-83.0) | **<0.001** |
| Age ≥70 years | 220 290 (52.2%) | 41 601 (66.0%) | **<0.001** |
| Female sex | 215 077 (51.0%) | 32 240 (51.2%) | 0.352 |
| **Cardiovascular risk factors*** | | | |
| Obesity | 40 770 (9.7%) | 4299 (6.8%) | <0.001 |
| Essential arterial hypertension | 200 379 (47.5%) | 25 074 (39.8%) | <0.001 |
| Hyperlipidaemia | 62 972 (14.9%) | 6763 (10.7%) | <0.001 |
| **VTE risk factors** | | | |
| Cancer | 84 975 (20.1%) | 17 384 (27.6%) | <0.001 |
| Surgery | 238 103 (56.4%) | 35 075 (55.7%) | <0.001 |
| Known thrombophilia | 6550 (1.6%) | 430 (0.7%) | <0.001 |
| Pregnancy | 565 (0.13%) | 22 (0.03%) | <0.001 |
| **Comorbidities** |  |  |  |
| Coronary artery disease | 53 976 (12.8%) | 10 378 (16.5%) | <0.001 |
| Heart failure | 91 214 (21.6%) | 21 676 (34.4%) | <0.001 |
| Peripheral artery disease | 11 637 (2.8%) | 3023 (4.8%) | <0.001 |
| Atrial fibrillation/flutter | 53 954 (12.8%) | 14 980 (23.8%) | <0.001 |
| Chronic obstructive pulmonary disease | 38 173 (9.0%) | 7277 (11.6%) | <0.001 |
| Acute or chronic renal failure | 89 690 (21.3%) | 26 725 (42.4%) | <0.001 |
| COVID-19 | 2398 (0.6%) | 964 (1.5%) | <0.001 |
| Diabetes mellitus | 75 111 (17.8%) | 14 809 (23.5%) | <0.001 |
| Anaemia | 36 394 (8.6%) | 7518 (11.9%) | <0.001 |
| **Clinical signs of PE severity** |  |  |  |
| **Severe pulmonary embolism** | **99 868 (23.7%)** | **37 318 (59.2%)** | **<0.001** |
| Syncope | 10 921 (2.6%) | 1437 (2.3%) | <0.001 |
| Tachycardia | 13 424 (3.2%) | 3713 (5.9%) | <0.001 |
| RV dysfunction | 85 681 (20.3%) | 29 861 (47.4%) | <0.001 |
| Haemodynamic instability | 13 548 (3.2%) | 39 673 (48.7) | <0.001 |
| Shock | 10 043 (2.4%) | 15 451 (24.5%) | <0.001 |
| Cardiopulmonary resuscitation | 5337 (1.3%) | 23 123 (36.7%) | <0.001 |
| **Treatment** | | | |
| Admission to intensive care unit | 71 329 (16.9%) | 20 985 (33.3%) | <0.001 |
| Mechanical ventilation | 14 185 (3.4%) | 4781 (7.6%) | <0.001 |
| Systemic thrombolysis | 10 723 (2.5%) | 9419 (15.0%) | <0.001 |
| Catheter-directed treatment | 1430 (0.3%) | 307 (0.5%) | <0.001 |
| Surgical embolectomy | 415 (0.1%) | 192 (0.3%) | <0.001 |
| **Adverse events during hospitalisation** | | | |
| Stroke | 11 648 (2.8%) | 4942 (7.8%) | <0.001 |
| Pneumonia | 110 691 (26.2%) | 19 672 (31.2%) | <0.001 |
| **Major bleeding** | **44 361 (10.5%)** | **17 524 (27.8%)** | **<0.001** |
| Intracerebral bleeding | 2260 (0.5%) | 1199 (1.9%) | <0.001 |
| Gastrointestinal bleeding | 6282 (1.5%) | 2429 (3.9%) | <0.001 |
| Haemarthros | 105 (0.1%) | 11 (0.1%) | 0.332 |
| Transfusion of blood constituents | 39 403 (9.3%) | 15 728 (25.0%) | <0.001 |
| **Duration of hospitalisation** | | | |
| Days in hospital, *median (IQR)* | 8.0 (5.0-14.0) | 5.0 (1.0-14.0) | <0.001 |
| Hospitalisation >7 days | 227 668 (54.0%) | 254 360 (42.4%) | <0.001 |
| Hospitalisation >10 days | 154 470 (36.6%) | 21 019 (33.4%) | <0.001 |
| **Hospitalisation costs** | | | |
| Hospital reimbursement, *median (IQR)* | 3570 (2972-5290) | 4472 (1488-11,346) | <0.001 |
| Hospital reimbursement, *mean ± SD* | 6724 ± 13 274 | 11 035 ± 20 367 | <0.001 |

* Coding of the chronic cardiovascular risk factors listed in the Table encompasses a broad spectrum of the degree/severity of clinical or laboratory abnormalities, and this fact may have exerted confounding effects on their association with in-hospital mortality in the present study.

COVID-19, coronavirus disease 19; IQR, interquartile range; MACCE, major adverse cardiac and cerebrovascular events, defined as all-cause in-hospital death, acute myocardial infarction, or stroke; PE, pulmonary embolism; RV, right ventricle; SD, standard deviation; VTE, venous thromboembolism.

**Table S2 Outcomes and hospitalisation costs of 137 186 patients with acute severe pulmonary embolism in the years 2016-2020, stratified by systemic thrombolytic therapy**

| **Parameters** | **No systemic thrombolysis**  **(*n*=121 136; 88.3%)** | **Received systemic thrombolysis**  **(*n*=16 050; 11.7%)** | **p value** |
| --- | --- | --- | --- |
| **Length of hospital stay** | | | |
| Days in hospital, *median (IQR)* | 9.0 (5.0-16.0) | 7.0 (2.0-14.0) | <0.001 |
| Hospitalisation >7 days | 70 782 (58.4%) | 7589 (47.3%) | <0.001 |
| Hospitalisation >10 days | 50 495 (41.7%) | 5455 (34.0%) | <0.001 |
| **Adverse events during hospitalisation** | | | |
| In-hospital death | 29 373 (24.2%) | 7945 (49.5%) | <0.001 |
| Major bleeding | 21 650 (17.9%) | 3863 (24.1%) | <0.001 |
| Intracranial bleeding | 1017 (0.8%) | 284 (1.8%) | <0.001 |
| Gastrointestinal bleeding | 2976 (2.5%) | 400 (2.5%) | 0.785 |
| Transfusion of erythrocyte concentrates | 19 838 (16.4%) | 3506 (21.8%) | <0.001 |
| **Hospitalisation costs** | | | |
| Median reimbursed costs, *euro (IQR)* | 3622 (3491-7803) | 3600 (2670-9610) | <0.001 |
| Mean reimbursed costs, euro ± SD | 10 008 ± 20261 | 9928 ± 18 197 | <0.001 |
| Hospitalisation costs >10 000 euro | 24 860 (20.5%) | 3924 (24.4%) | <0.001 |

IQR, interquartile range; SD, standard deviation.

**Table S3 Outcomes and hospitalisation costs of 137 186 patients with acute severe pulmonary embolism in the years 2016-2020, stratified by surgical embolectomy**

| **Parameters** | **No surgical embolectomy**  **(*n*=136,682; 99.6%)** | **Underwent surgical embolectomy**  **(*n*=504; 0.4%)** | **p value** |
| --- | --- | --- | --- |
| **Length of hospital stay** | | | |
| Days in hospital, *median (IQR)* | 9.0 (5.0-16.0) | 15.0 (8.0-27.8) | <0.001 |
| Hospitalisation >7 days | 77 991 (57.1%) | 380 (75.4%) | <0.001 |
| Hospitalisation >10 days | 55 615 (40.7%) | 335 (66.5%) | <0.001 |
| **Adverse events during hospitalisation** | | | |
| In-hospital death | 37 137 (27.2%) | 181 (35.9%) | <0.001 |
| Major bleeding | 25 067 (18.3%) | 446 (88.5%) | <0.001 |
| Intracranial bleeding | 1294 (0.9%) | 7 (1.4%) | 0.348 |
| Gastrointestinal bleeding | 3362 (2.5%) | 14 (2.8%) | 0.645 |
| Transfusion of erythrocyte concentrates | 22 899 (16.8%) | 445 (88.3%) | <0.001 |
| **Hospitalisation costs** | | | |
| Median reimbursed costs, *euro (IQR)* | 3622 (3101-7884) | 27 461 (14 384 - 44 931) | <0.001 |
| Mean reimbursed costs, euro ± SD | 9908 ± 19 905 | 34 707 ± 34 049 | <0.001 |
| Hospitalisation costs >10.000 euro | 28 364 (20.8%) | 420 (83.3%) | <0.001 |

IQR, interquartile range; SD, standard deviation.

**Table S4 Outcomes and hospitalisation costs of 137 186 patients with acute severe pulmonary embolism in the years 2016-2020, stratified by catheter-directed treatment**

| **Parameters** | **No CDT**  **(*n*=135 805; 99.0%)** | **Underwent CDT**  **(*n*=1381; 1.0%)** | **p value** |
| --- | --- | --- | --- |
| **Length of hospital stay** | | | |
| Days in hospital, *median (IQR)* | 9.0 (5.0-16.0) | 8.0 (5.0-15.0) | 0.249 |
| Hospitalisation >7 days | 77 622 (57.2%) | 749 (54.2%) | 0.029 |
| Hospitalisation >10 days | 55 441 (40.8%) | 509 (36.9%) | 0.003 |
| **Adverse events during hospitalisation** | | | |
| In-hospital death | 37 039 (27.3%) | 279 (20.2%) | <0.001 |
| Major bleeding | 25 133 (18.5%) | 380 (27.5%) | <0.001 |
| Intracranial bleeding | 1271 (0.9%) | 30 (2.2%) | <0.001 |
| Gastrointestinal bleeding | 3339 (2.5%) | 37 (2.7%) | 0.599 |
| Transfusion of erythrocyte concentrates | 22 995 (16.9%) | 349 (25.3%) | <0.001 |
| **Hospitalisation costs** | | | |
| Median reimbursed costs, *euro (IQR)* | 3618 (3085-7824) | 8384 (7028 - 10 381) | <0.001 |
| Mean reimbursed costs, euro ± SD | 9960 ± 20 028 | 13 803 ± 19 895 | <0.001 |
| Hospitalisation costs >10 000 euro | 28 190 (20.8%) | 594 (43.0%) | <0.001 |

CDT, catheter-directed treatment; IQR, interquartile range; SD, standard deviation.

**Table S5** **Outcomes and hospitalisation costs of 25 494 patients with acute pulmonary embolism and shock in the years 2016-2020, stratified by systemic thrombolytic therapy**

| **Parameters** | **No systemic thrombolysis**  **(*n*=19 540; 76.6%)** | **Received systemic thrombolysis**  **(*n*=5954; 23.4%)** | **p value** |
| --- | --- | --- | --- |
| **Length of hospital stay** | | | |
| Days in hospital, *median (IQR)* | 14.0 (4.0-30.0) | 6.0 (1.0-15.0) | <0.001 |
| Hospitalisation >7 days | 12 715 (65.1%) | 2645 (44.4%) | <0.001 |
| Hospitalisation >10 days | 11 278 (57.7%) | 2092 (35.1%) | <0.001 |
| **Adverse events during hospitalisation** | | | |
| In-hospital death | 11 456 (58.6%) | 3995 (67.1%) | <0.001 |
| Major bleeding | 10 317 (52.8%) | 2130 (35.8%) | <0.001 |
| Intracranial bleeding | 369 (1.9%) | 112 (1.9%) | 0.971 |
| Gastrointestinal bleeding | 1335 (6.8%) | 243 (4.1%) | <0.001 |
| Transfusion of erythrocyte concentrates | 9911 (50.7%) | 1991 (33.4%) | <0.001 |
| **Hospitalisation costs** | | | |
| Median reimbursed costs, *euro (IQR)* | 12 436 (3883 - 33 972) | 4675 (1535 - 15 444) | <0.001 |
| Mean reimbursed costs, euro ± SD | 26 200 ± 37 895 | 13 118 ± 21 736 | <0.001 |
| Hospitalisation costs >10 000 euro | 10 933 (56.0%) | 2041 (34.3%) | <0.001 |

IQR, interquartile range; SD, standard deviation.

**Table S6 Outcomes and hospitalisation costs of 25 494 patients with acute pulmonary embolism and shock in the years 2016-2020, stratified by surgical embolectomy**

| **Parameters** | **No surgical embolectomy**  **(*n*=25 302; 99.2%)** | **Underwent surgical embolectomy**  **(*n*=192; 0.8%)** | **p value** |
| --- | --- | --- | --- |
| **Length of hospital stay** | | | |
| Days in hospital, *median (IQR)* | 11.0 (2.0-26.0) | 15.0 (5.0-29.0) | <0.001 |
| Hospitalisation >7 days | 15 227 (60.2%) | 133 (69.3%) | 0.010 |
| Hospitalisation >10 days | 13 246 (52.4%) | 124 (64.6%) | 0.001 |
| **Adverse events during hospitalisation** | | | |
| In-hospital death | 15 342 (60.6%) | 109 (56.8%) | 0.275 |
| Major bleeding | 12 265 (48.5%) | 182 (94.8%) | <0.001 |
| Intracranial bleeding | 477 (1.9%) | 4 (2.1%) | 0.786 |
| Gastrointestinal bleeding | 1568 (6.2%) | 10 (5.2%) | 0.763 |
| Transfusion of erythrocyte concentrates | 11 721 (46.3%) | 181 (94.3%) | <0.001 |
| **Hospitalisation costs** | | | |
| Median reimbursed costs, *euro (IQR)* | 10 327 (3554 – 28 760) | 33 661 (15 926 – 49 207) | <0.001 |
| Mean reimbursed costs, euro ± SD | 22 996 ± 35 136 | 42 271 ± 42 272 | <0.001 |
| Hospitalisation costs >10 000 euro | 12 813 (50.6%) | 161 (83.9%) | <0.001 |

IQR, interquartile range; SD, standard deviation.

**Table S7 Outcomes and hospitalisation costs of 25 494 patients with acute pulmonary embolism and shock in the years 2016-2020, stratified by catheter-directed treatment**

| **Parameters** | **No CDT**  **(*n*=25 197; 98.8%)** | **Underwent CDT**  **(*n*=297; 1.2%)** | **p value** |
| --- | --- | --- | --- |
| **Length of hospital stay** | | | |
| Days in hospital, *median (IQR)* | 12.0 (2.0-26.0) | 8.0 (2.0-20.5) | 0.006 |
| Hospitalisation >7 days | 15 204 (60.3%) | 156 (52.5%) | 0.006 |
| Hospitalisation >10 days | 13 237 (52.5%) | 133 (44.8%) | 0.008 |
| **Adverse events during hospitalisation** | | | |
| In-hospital death | 15 284 (60.7%) | 167 (56.2%) | 0.120 |
| Major bleeding | 12 279 (48.7%) | 168 (56.6%) | 0.007 |
| Intracranial bleeding | 468 (1.9%) | 13 (4.4%) | 0.002 |
| Gastrointestinal bleeding | 1567 (6.2%) | 11 (3.7%) | 0.074 |
| Transfusion of erythrocyte concentrates | 11 741 (46.6%) | 161 (54.2%) | 0.009 |
| **Hospitalisation costs** | | | |
| Median reimbursed costs, *euro (IQR)* | 10 436 (3554 – 29 108) | 10 041 (5205-25 272) | 0.105 |
| Mean reimbursed costs, euro ± SD | 23 164 ± 35 294 | 21 103 ± 29 575 | 0.105 |
| Hospitalisation costs >10 000 euro | 12 820 (50.9%) | 154 (51.9%) | 0.739 |

CDT, catheter-directed treatment; IQR, interquartile range; SD, standard deviation.

**Fig. S1** Mean costs for PE-related hospitalisations stratified by Charlson comorbidity index severity class over the entire study period (2016-2020).

**Fig. S2** Mean costs for PE-related hospitalisations in euro in hospitals of rural, suburban and urban areas throughout the study period (2016-2020).


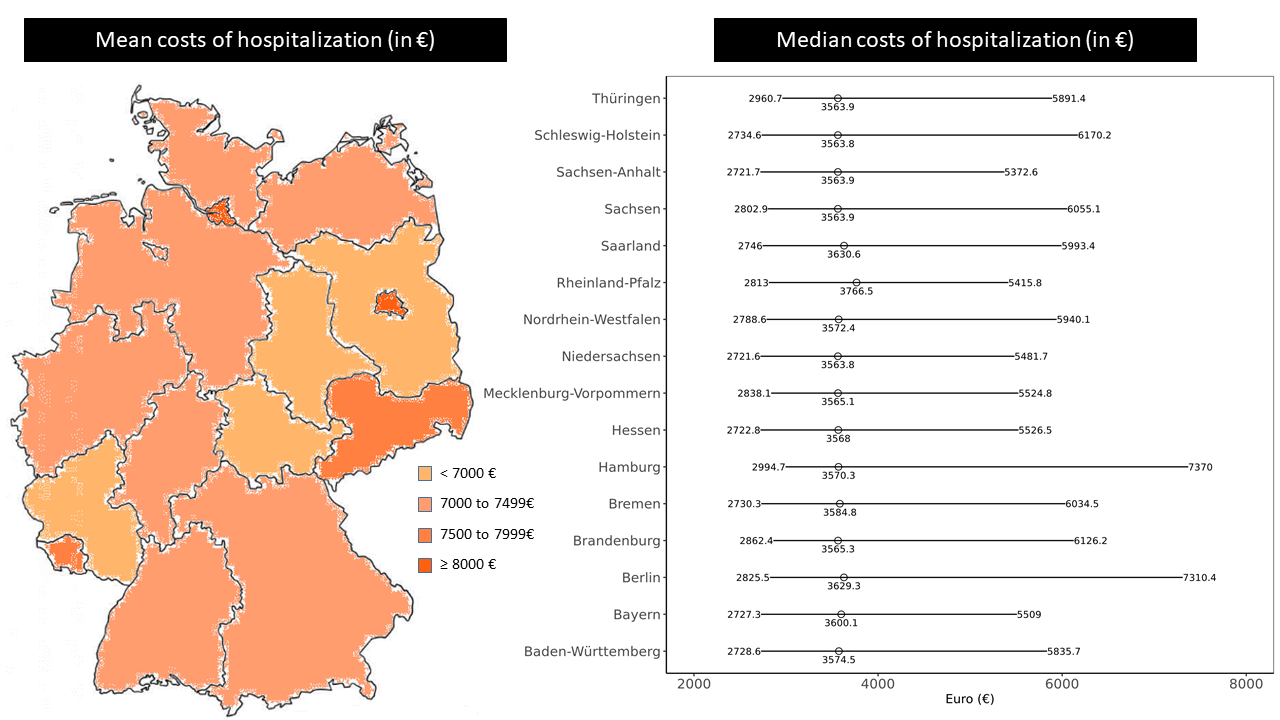


**Fig. S3** Range of mean costs (left panel), and median costs with the corresponding interquartile range (right panel), for PE-related hospitalisations per federal state (Bundesland) in Germany during the study period.

**
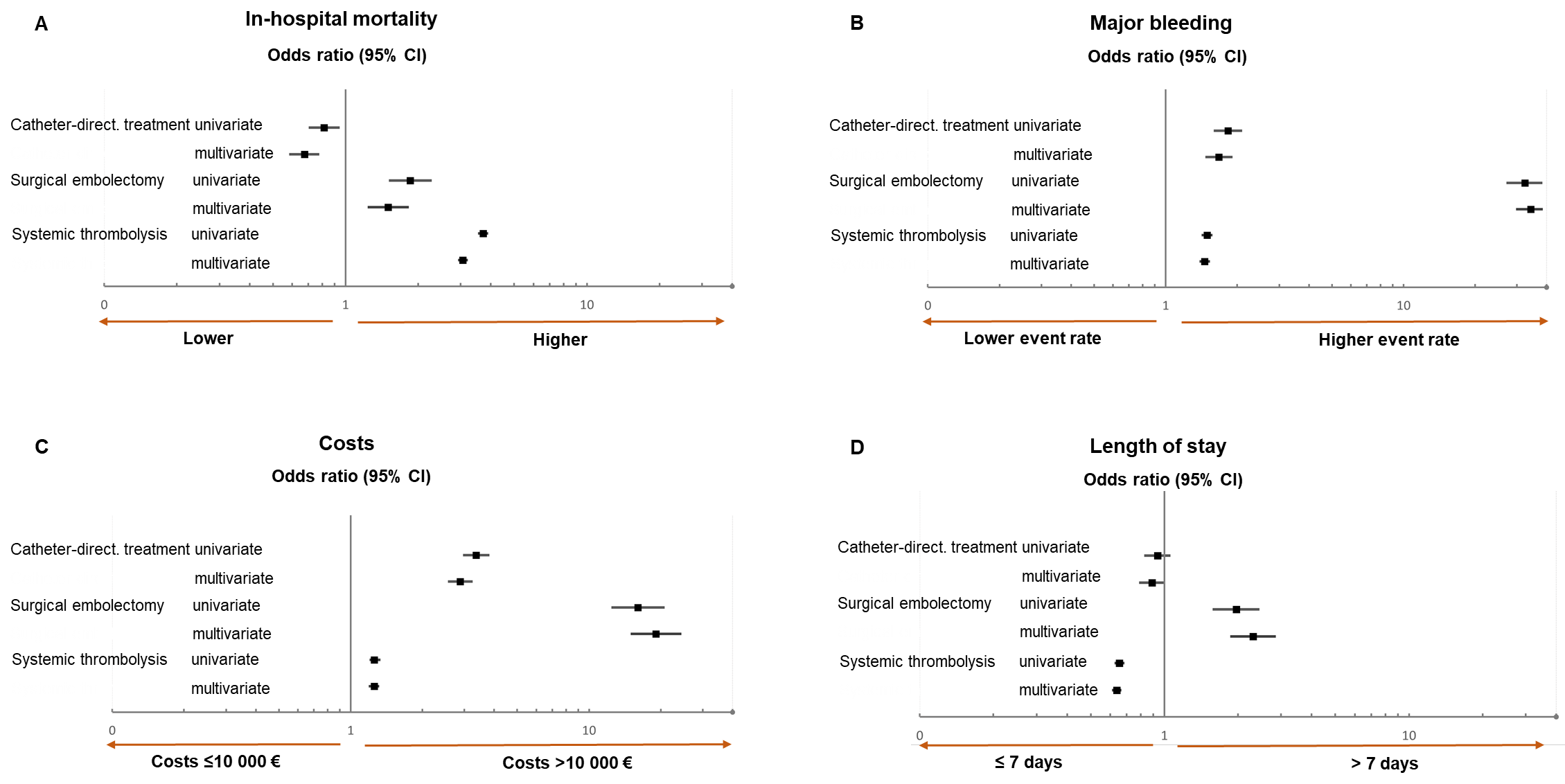
**

**Fig. S4** Association, in patients with severe pulmonary embolism, of different reperfusion treatment procedures with in-hospital mortality (Panel A), major bleeding (Panel B), reimbursed hospitalisation costs (Panel C) and length of hospital stay >7 days (Panel D). Results of univariate and multivariable logistic regression analysis are presented as Odds Ratios (OR) with corresponding 95% confidence intervals (CI), representing the use versus non-use of the respective treatment. The multivariable model adjusted for the following variables: age, sex, obesity, diabetes mellitus, cancer, oronary artery disease, heart failure, chronic obstructive pulmonary disease, essential arterial hypertension, acute/chronic kidney failure, surgery, chronic anaemia, and atrial fibrillation/flutter.
